# Supplementary material for: Industry involvement in evidence production for genomic medicine: A bibliometric and funding analysis of decision impact studies
Source: PLoS One. 2023 Apr 27;18(4):e0285122. doi: 10.1371/journal.pone.0285122 (PMC10138259; doi:10.1371/journal.pone.0285122)
Supplement: S1 Appendix — (DOCX) [file pone.0285122.s001.docx]

**S1 Appendix. Full Electronic Search Strategy for Scopus Database**

**Scopus Search Strategy:**

ALL("decision impact" or "decision-impact" or "decision-making impact" or "decision making impact") AND ( EXCLUDE ( DOCTYPE,"ch" ) OR EXCLUDE ( DOCTYPE,"bk" ) OR EXCLUDE ( DOCTYPE,"no" ) OR EXCLUDE ( DOCTYPE,"ed" ) OR EXCLUDE ( DOCTYPE,"le" ) OR EXCLUDE ( DOCTYPE,"sh" ) ) AND ( EXCLUDE ( LANGUAGE,"Spanish" ) OR EXCLUDE ( LANGUAGE,"French" ) OR EXCLUDE ( LANGUAGE,"German" ) OR EXCLUDE ( LANGUAGE,"Portuguese" ) OR EXCLUDE ( LANGUAGE,"Chinese" ) OR EXCLUDE ( LANGUAGE,"Japanese" ) OR EXCLUDE ( LANGUAGE,"Persian" ) OR EXCLUDE ( LANGUAGE,"Italian" ) OR EXCLUDE ( LANGUAGE,"Czech" ) )
