# Supplementary material for: Industry involvement in evidence production for genomic medicine: A bibliometric and funding analysis of decision impact studies
Source: PLoS One. 2023 Apr 27;18(4):e0285122. doi: 10.1371/journal.pone.0285122 (PMC10138259; doi:10.1371/journal.pone.0285122)
Supplement: S4 Appendix — (DOCX) [file pone.0285122.s004.docx]

**S4 Appendix. R-Code Used to Identify Title Topics**

data <-read.csv("titles.csv", header = TRUE)

library(tm)

library(ggplot2)

library(wordcloud)

library(RWeka)

mycorpus <- Corpus(VectorSource(data$TI))

#Text Cleaning

# Convert the text to lower case

mycorpus <- tm_map(mycorpus, content_transformer(tolower))

# Remove numbers

mycorpus <- tm_map(mycorpus, removeNumbers)

# Remove english common stopwords

mycorpus <- tm_map(mycorpus, removeWords, stopwords("english"))

# Remove punctuations

mycorpus <- tm_map(mycorpus, removePunctuation)

# Eliminate extra white spaces

mycorpus <- tm_map(mycorpus, stripWhitespace)

as.character(mycorpus[[1]])

#Bi-Grams

minfreq_bigram<-5

token_delim <- " \\t\\r\\n.!?,;\"()"

bitoken <- NGramTokenizer(mycorpus, Weka_control(min=2,max=2, delimiters = token_delim))

two_word <- data.frame(table(bitoken))

sort_two <- two_word[order(two_word$Freq,decreasing=TRUE),]

wordcloud(sort_two$bitoken,sort_two$Freq,random.order=FALSE,scale = c(2,0.35),min.freq = minfreq_bigram,colors = brewer.pal(8,"Dark2"),max.words=150)
