# Supplementary material for: Industry involvement in evidence production for genomic medicine: A bibliometric and funding analysis of decision impact studies
Source: PLoS One. 2023 Apr 27;18(4):e0285122. doi: 10.1371/journal.pone.0285122 (PMC10138259; doi:10.1371/journal.pone.0285122)
Supplement: S1 Table — (DOCX) [file pone.0285122.s005.docx]

**S1 Table. Table of included publications**

| **No.** | **First Author** | **Title** | **Year** | **Journal** | **Included in Bibliometric Analysis** | **Included in Funding Analysis** |
| --- | --- | --- | --- | --- | --- | --- |
| 1 | Akerley, W. | The Impact of a Serum Based Proteomic Mass Spectrometry Test on Treatment Recommendations in Advanced Non-Small-Cell Lung Cancer | 2013 | Curr Med Res Opin | X | X |
| 2 | Albanell, J. | Pooled Analysis of 4 European Studies Assessing the Impact of Oncotype Dx (R) on Treatment Decisions | 2013 | Breast | X |  |
| 3 | Albanell, J. | Pooled Analysis of Prospective European Studies Assessing the Impact of Using The 21-Gene Recurrence Score Assay on Clinical Decision Making in Women with Oestrogen Receptor-Positive, Human Epidermal Growth Factor Receptor 2-Negative Early-Stage Breast Cancer | 2016 | Eur J Cancer | X | X |
| 4 | Anastasiadou, L. | Oncotype Dx (R)-Remar (Rhein-Main-Registry)-Study: Use of the Oncotype Dx (R) Assay in Early Breast Cancer in Certified Breast Cancer Centers in Rhine-Main Region, Germany | 2018 | Cancer Res | X | X |
| 5 | Anders, S. | Correlation And Outcome Prediction of Biomarkers in Early, Estrogen Receptor-Positive and Her2-Negative Breast Cancer: Comparison of Endopredict, UPA/PAI-1 And Ki67 | 2017 | Int J Gynecol Cancer | X | X |
| 6 | Ando, K. | Sunrise-Di Study: Decision Impact of the 12-Gene Rs Assay on Adjuvant Chemotherapy for Stage II And III A/B Colon Cancer | 2019 | Ann Oncol | X | X |
| 7 | Antoine, E. | Paradygm: Impact of 21 Genes Recurrence Score Assay (ODx) on Final Decision and Heterogeneity of Decisions Between Different Tumor Boards | 2018 | Eur J Cancer | X | X |
| 8 | Arnell, C. | Guided Therapy Selection in Rheumatoid Arthritis Using a Molecular Signature Response Classifier: An Assessment of Budget Impact and Clinical Utility | 2021 | J Manag Care Spec Ph | X | X |
| 9 | Augustovski, F. | Response To Real-Life Decision-Making Impact of Oncotype Dx | 2015 | Breast Cancer Res Tr | X | X |
| 10 | Augustovski, F. | Meta-Analysis of Decision Impact and Net Decision Change in Adjuvant Chemotherapy Allocation in Early-Stage Node-Negative, Estrogen Receptor-Positive Breast Cancer With a 21-Gene Assay | 2014 | Value Health | X |  |
| 11 | Augustovski, F. | Decision-Making Impact on Adjuvant Chemotherapy Allocation in Early Node-Negative Breast Cancer with A 21-Gene Assay: Systematic Review and Meta-Analysis | 2015 | Breast Cancer Res Tr | X |  |
| 12 | Badani, K. | Effect of a Genomic Classifier Test on Clinical Practice Decisions for Patients with High-Risk Prostate Cancer After Surgery | 2015 | Bju Int | X | X |
| 13 | Badani, K. | Impact of a Genomic Classifier of Metastatic Risk on Postoperative Treatment Recommendations for Prostate Cancer Patients: A Report from the Decide Study Group | 2013 | Oncotarget | X | X |
| 14 | Bargallo-Rocha, J. | A Study of The Impact of the 21-Gene Breast Cancer Assay on the Use of Adjuvant Chemotherapy in Women with Breast Cancer in a Mexican Public Hospital | 2012 | Ann Oncol | X |  |
| 15 | Bargallo-Rocha, J. | A Study of the Impact of the 21-Gene Breast Cancer Assay on the Use of Adjuvant Chemotherapy in Women with Breast Cancer in a Mexican Public Hospital | 2015 | J Surg Oncol | X | X |
| 16 | Bargallo-Rocha, J. | Cost-Effectiveness Analysis of the 21-Gene Breast Cancer Test in Mexico | 2013 | Breast | X |  |
| 17 | Barni, S. | Is the Oncotype Dx Test Useful in Elderly Breast Cancer Patients: A Subgroup Analysis of Real-Life Italian PONDx Study | 2022 | Breast Cancer Res Tr | X | X |
| 18 | Barni, S. | Value of Genomic Test (Oncotype Dx) in Elderly Patients: An Italian Survey | 2020 | Tumori | X |  |
| 19 | Barry, M. | A Prospective Decision Impact Study to Evaluate the Utility of the Oncotype Dx Breast DCIS Score Assay in Selecting Patients with Ductal Carcinoma In Situ (DCIS) Following Breast Conservation Surgery (BCS) for Radiotherapy | 2022 | J Clin Oncol | X | X |
| 20 | Berdunov, V. | Budget Impact Analysis of the Oncotype Dx Breast Cancer Recurrence Score Test to Guide Chemotherapy Use in Er+/Her2-Node-Negative Early Invasive Breast Cancer | 2021 | Value Health | X | X |
| 21 | Bertelli, G. | Real-Life Decision-Making Impact of Oncotype Dx | 2015 | Breast Cancer Res Tr | X |  |
| **No.** | **First Author** | **Title** | **Year** | **Journal** | **Included in Bibliometric Analysis** | **Included in Funding Analysis** |
| 22 | Blohmer, J. | German Multicentre Decision Impact Study of Oncotype Dx Recurrence Score (Rs) on Adjuvant Treatment in Estrogen Receptor Positive (Er Plus) Node Negative (N0) and Node Positive (N Plus) Early Breast Cancer | 2011 | Breast | X |  |
| 23 | Blohmer, J. | Using the 21-Gene Assay to Guide Adjuvant Chemotherapy Decision-Making in Early-Stage Breast Cancer: A Cost-Effectiveness Evaluation in the German Setting | 2013 | J Med Econ | X |  |
| 24 | Blohmer, J. | Impact of the Oncotype Dx (R) Recurrence Score (R) Assay on Therapy Recommendations for Er-Positive (Er+), Node Negative (N0) And Node Positive (N+) Early Breast Cancer - Results of an Interim Analysis of the German Decision Impact Study | 2011 | Onkologie | X |  |
| 25 | Blok, E. | Systematic Review of the Clinical and Economic Value of Gene Expression Profiles for Invasive Early Breast Cancer Available in Europe | 2018 | Cancer Treat Rev | X | X |
| 26 | Blumenthal, D. | Clinical Utility and Treatment Outcome of Comprehensive Genomic Profiling in High Grade Glioma Patients | 2016 | J Neuro-Oncol | X | X |
| 27 | Bremer, T. | Interim Analysis of the Dcisionrt Predict Study: Clinical Utility of a Biologic Signature Predictive of Radiation Therapy Benefit in Patients With DCIS | 2019 | Ann Surg Oncol | X | X |
| 28 | Brenner, B. | Impact of the 12-Gene Colon Cancer Assay on Clinical Decision Making for Adjuvant Therapy In Stage II Colon Cancer Patients | 2016 | Value Health | X | X |
| 29 | Brufsky, A. | Predictive And Prognostic Value of the 21-Gene Recurrence Score in Hormone Receptor-Positive, Node-Positive Breast Cancer | 2014 | Am J Clin Oncol-Canc | X | X |
| 30 | Burke, E. | The 21-Gene Breast Cancer Assay: A Roadmap of Clinical Evidence | 2014 | Eur J Cancer | X | X |
| 31 | Chao, C. | Clinical Development of Gene Expression Assay (Oncotype Dx) And Future Challenges in a ctDNA-Based Test Era | 2021 | Ann Oncol | X | X |
| 32 | Cheung, P. | Initial Experience with the Oncotype Dx Assay in Decision-Making for Adjuvant Therapy of Early Oestrogen Receptor-Positive Breast Cancer in Hong Kong | 2014 | Hong Kong Med J | X | X |
| 33 | Chin-Lenn, L. | The Impact and Indications for Oncotype Dx on Adjuvant Treatment Recommendations When Third-Party Funding is Unavailable | 2018 | Asia-Pac J Clin Onco | X | X |
| 34 | Chin-Lenn, L. | Indications for, and Impact of Oncotype Dx on Adjuvant Treatment Recommendations When Third Party Funding is Unavailable | 2016 | Cancer Res | X |  |
| 35 | Cognetti, F. | Pondx: Real-Life Utilization and Decision Impact of the 21-Gene Assay on Clinical Practice in Italy | 2021 | Npj Breast Cancer | X | X |
| 36 | Coquerelle, S. | Impact of Next Generation Sequencing on Clinical Practice in Oncology in France: Better Genetic Profiles for Patients Improve Access to Experimental Treatments | 2020 | Value Health | X | X |
| 37 | Coquerelle, S. | Next Generation Sequencing (NGS) And Patient Pathways: What Is the Impact on Clinical Decision? | 2018 | Ann Oncol | X |  |
| 38 | Curtit, E. | Results Of Pondx, A Prospective Multicenter Study of The Oncotype Dx (R) Breast Cancer Assay: Real-Life Utilization and Decision Impact in French Clinical Practice | 2019 | Breast | X | X |
| 39 | Dabbs, D. | A Comparison of Breast Cancer Multianalyte Assays with Algorithmic Analyses (MAAA) For Their Net Predictive/Prognostic Value | 2015 | Clin Adv Hematol Onc | X | X |
| 40 | Davey, M. | Is Radiomic MRI a Feasible Alternative to Oncotypedx (R) Recurrence Score Testing? A Systematic Review and Meta-Analysis | 2021 | Bjs Open | X | X |
| 41 | De Boer, R. | The Impact of a Genomic Assay (Oncotype Dx) on Adjuvant Treatment Recommendations in Early Breast Cancer | 2013 | Med J Australia | X | X |
| 42 | De Boer, R. | Australian Decision Impact Study: The Impact of Oncotype Dx Recurrence Score (RS) on Adjuvant Treatment Decisions in Hormone Receptor Positive (HR Plus), Node Negative (N0) And Node Positive (N Plus) Early Stage Breast Cancer (ESBC) in the Multidisciplinary Clinic (MDC). | 2011 | Cancer Res | X |  |
| **No.** | **First Author** | **Title** | **Year** | **Journal** | **Included in Bibliometric Analysis** | **Included in Funding Analysis** |
| 43 | De Lima, L. | Cost-Benefit Analysis of a 21-Gene Recurrence Score for Early Stage Breast Cancer In Singapore | 2010 | Value Health | X |  |
| 44 | Degtiar, I. | A Prospective Registry Study Assessing Decision Impact and Patient Outcomes Following Gene-Expression Profiling for Tumor-Site Origin. | 2013 | J Clin Oncol | X | X |
| 45 | Dieci, M. | Impact of 21-Gene Breast Cancer Assay on Treatment Decision for Patients with T1-T3, N0-N1, Estrogen Receptor-Positive/Human Epidermal Growth Receptor 2-Negative Breast Cancer: Final Results of the Prospective Multicenter Roxane Study | 2019 | Oncologist | X | X |
| 46 | Dillon, M. | Evolution of the Post-Surgical Breast Cancer Pathway for Adjuvant Treatments Following Introduction of Genomic Profiling for Selected Women with Hormone Receptor Positive, Her2 Negative Disease | 2020 | Ejso-Eur J Surg Onc | X | X |
| 47 | Eichler, C. | Gene-Expression Profiling - A Decision Impact Analysis: Decision Dependency on Oncotype Dx as a Function of Oncological Work Experience in 117 Cases | 2019 | Anticancer Res | X | X |
| 48 | Eiermann, W. | The 21-Gene Recurrence Score Assay Impacts Adjuvant Therapy Recommendations for Er-Positive, Node-Negative and Node-Positive Early Breast Cancer Resulting in A Risk-Adapted Change in Chemotherapy Use | 2013 | Ann Oncol | X | X |
| 49 | Epelbaum, R. | Molecular Profiling (MP)-Selected Therapy for the Treatment of Patients with Advanced Pancreaticobiliary Cancer (PBC) | 2013 | J Clin Oncol | X |  |
| 50 | Epelbaum, R. | Molecular Profiling-Selected Therapy for Treatment of Advanced Pancreaticobiliary Cancer: A Retrospective Multicenter Study | 2015 | Biomed Res Int | X | X |
| 51 | Esin, E. | Prosigna Assay for Treatment Decisions in Early Breast Cancer: A Single Center, Decision Impact Study | 2019 | Cancer Res | X | X |
| 52 | Ettl, J. | Decision Impact and Feasibility of Different Asco-Recommended Biomarkers in Early Breast Cancer: Prospective Comparison of Molecular Marker Endopredict and Protein Marker UPA/PAI-1 | 2017 | Plos One | X | X |
| 53 | Fallowfield, L. | Enhancing Decision-Making About Adjuvant Chemotherapy in Early Breast Cancer Following Endopredict Testing | 2018 | Psycho-Oncology | X | X |
| 54 | Ferguson, J. | Impact of a Bronchial Genomic Classifier on Clinical Decision Making in Patients Undergoing Diagnostic Evaluation for Lung Cancer | 2016 | Bmc Pulm Med | X | X |
| 55 | Gligorov, J. | The 21-Gene Assay in the Decision Impact Assessment of Er+, Her2-Breast Cancer: A French Real Life Prospective Study | 2017 | Cancer Res | X | X |
| 56 | Gligorov, J. | Prospective Clinical Utility Study of the Use of the 21-Gene Assay in Adjuvant Clinical Decision Making in Women with Estrogen Receptor-Positive Early Invasive Breast Cancer: Results from the Switch Study | 2015 | Oncologist | X | X |
| 57 | Gomez, H. | Practice-Changing Use of the 21-Gene Test for the Management of Patients with Early-Stage Breast Cancer in Latin America | 2021 | Jco Glob Oncol | X | X |
| 58 | Harnan, S. | Tumour Profiling Tests to Guide Adjuvant Chemotherapy Decisions in Early Breast Cancer: A Systematic Review and Economic Analysis | 2019 | Health Technol Asses | X | X |
| 59 | Hay, M. | Identifying Opportunities and Challenges for Patients with Sarcoma as a Result of Comprehensive Genomic Profiling of Sarcoma Specimens | 2020 | Jco Precis Oncol | X | X |
| 60 | Henner, W. | Estimation of Expected Survival Time Using Gene Expression Profiling for Tumor Site Origin | 2012 | J Clin Oncol | X | X |
| 61 | Hequet, D. | Prospective, Multicenter French Study Evaluating the Clinical Impact of the Breast Cancer Intrinsic Subtype-Prosigna (R) Test in the Management of Early-Stage Breast Cancers | 2017 | Plos One | X | X |
| 62 | Hequet, D. | Prosigna (R) Test in Clinical Routine: Impact on Adjuvant Chemotherapy Decision and Medico-Economic Considerations in France | 2020 | Cancer Res | X |  |
| 63 | Hequet, D. | Prosigna Test in Breast Cancer: Real-Life Experience | 2021 | Breast Cancer Res Tr | X | X |
| **No.** | **First Author** | **Title** | **Year** | **Journal** | **Included in Bibliometric Analysis** | **Included in Funding Analysis** |
| 64 | Hogarth, D. | The Percepta Registry: A Prospective Registry to Evaluate Percepta Bronchial Genomic Classifier Patient Data | 2016 | Chest | X | X |
| 65 | Holt, S. | A Decision Impact, Decision Conflict and Economic Assessment of Routine Oncotype Dx Testing of 146 Women with Node-Negative or pNImi, ER-Positive Breast Cancer in the UK | 2013 | Brit J Cancer | X | X |
| 66 | Hornberger, J. | Meta-Analysis of the Decision Impact of the 21-Gene Breast Cancer Recurrence Score (R) in Clinical Practice | 2011 | Breast | X | X |
| 67 | Hornberger, J. | Meta-Analysis of the Decision Impact of the 21-Gene Breast Cancer Recurrence Score in Clinical Practice | 2010 | Cancer Res | X |  |
| 68 | Hornberger, J. | Economics of a Multi-Gene Assay to Predict Recurrence of Early Stage Breast Cancer: Experience of a Large United States Insurance Program | 2011 | Value Health | X | X |
| 69 | Hornberger, J. | Us Insurance Program's Experience with a Multigene Assay for Early-Stage Breast Cancer | 2011 | J Oncol Pract | X | X |
| 70 | Hornberger, J. | Us Insurance Program's Experience with a Multigene Assay for Early-Stage Breast Cancer | 2011 | Am J Manag Care | X |  |
| 71 | Igari, F. | The Applications of Plasma Cell-Free DNA in Cancer Detection: Implications in the Management of Breast Cancer Patients | 2022 | Crit. Rev. Oncol. Hematol. | X | X |
| 72 | Jaafar, C. | Impact of Oncotype Dx Testing on Adjuvant Treatment Decisions in Patients with Early Breast Cancer: A Single-Center Study in the United Arab Emirates | 2014 | Asia-Pac J Clin Onco | X | X |
| 73 | Jank, P. | Comparison of Risk Assessment in 1652 Early Er Positive, Her2 Negative Breast Cancer in a Real-World Data Set: Classical Pathological Parameters vs. 12-Gene Molecular Assay (Endopredict) | 2022 | Breast Cancer Res. Treat. | X | X |
| 74 | Katz, G. | Economic Impact of Gene Expression Profiling in Patients with Early-Stage Breast Cancer in France | 2015 | Plos One | X | X |
| 75 | Keegan, N. | The Impact of the 21 Gene Recurrence Score (RS) on Chemotherapy Prescribing in Estrogen Receptor (ER) Positive, Lymph Node Positive Early Stage Breast Cancer in Ireland | 2017 | Cancer Res | X | X |
| 76 | Kuchel, A. | The Impact of the 21-Gene Assay on Adjuvant Treatment Decisions in Oestrogen Receptor-Positive Early Breast Cancer: A Prospective Study | 2016 | Brit J Cancer | X | X |
| 77 | Kummel, S. | The Oncotype Dx Recurrence Score Assay Impacts Adjuvant Therapy Recommendations for Er-Positive (ER+), Node Negative (N0) and Node Positive (N+) Early Breast Cancer-Final Results of the German Decision Impact Study | 2012 | J Cancer Res Clin Oncol | X | X |
| 78 | Lasky, J. | The Impact of the Envisia Genomic Classifier in the Diagnosis and Management of Patients with Idiopathic Pulmonary Fibrosis | 2022 | Ann Am Thorac Soc | X | X |
| 79 | Lasky, J. | The Impact of the Envisia Genomic Classifier in the Diagnosis and Management of Patients with Idiopathic Pulmonary Fibrosis. | 2021 | Ann Am Thorac Soc | X |  |
| 80 | Lasky, J. | The Impact of the Envisia Genomic Classifier in the Diagnosis and Management of Patients with Interstitial Lung Disease | 2021 | Chest | X | X |
| 81 | Lerebours, F. | DI Study: Decision Impact of the Nanostring Technologies Prosigna (TM) in Early Breast Cancers | 2016 | Cancer Res | X | X |
| 82 | Levasseur, N. | Impact of the 21-Gene Recurrence Score Assay on the Treatment of Estrogen Receptor-Positive, Her2-Negative, Breast Cancer Patients With 1-3 Positive Nodes: A Prospective Clinical Utility Study | 2022 | Clin Breast Cancer | X | X |
| 83 | Lopes, G. | Cost-Benefit Analysis of a 21-Gene Recurrence Score for Early Stage Breast Cancer in Singapore | 2011 | Breast | X | X |
| 84 | Lux, M. | Budget Impact Analysis of Gene Expression Tests to Aid Therapy Decisions for Breast Cancer Patients in Germany | 2018 | Breast | X | X |
| 85 | Mamounas, E. | Chemotherapy (Ct) Decision in Node-Positive (N Plus), ER Plus, Early Breast Cancer (EBC) After New ASCO Guideline - Evidence for the 21-Gene Recurrence Score (RS) Assay | 2017 | Breast | X | X |
| **No.** | **First Author** | **Title** | **Year** | **Journal** | **Included in Bibliometric Analysis** | **Included in Funding Analysis** |
| 86 | Manders, J. | The 12-Gene DCIS Score Assay: Impact on Radiation Treatment (XRT) Recommendations and Clinical Utility | 2016 | Cancer Res | X | X |
| 87 | Markopoulos, C. | Overview of the Use of Oncotype Dx (R) as an Additional Treatment Decision Tool in Early Breast Cancer | 2013 | Expert Rev Anticanc | X | X |
| 88 | Markopoulos, C. | Clinical Evidence Supporting Genomic Tests in Early Breast Cancer: Do all Genomic Tests Provide the Same Information? | 2017 | Ejso-Eur J Surg Onc | X | X |
| 89 | Martin, M. | Prospective Study of the Impact of the Prosigna (TM) Assay on Adjuvant Clinical Decision-Making in Women with Estrogen Receptor-Positive, Her2-Negative, Node-Negative Breast Cancer: A Geicam Study | 2015 | Cancer Res | X | X |
| 90 | Mattar, A. | Substantial Reduction in Adjuvant Chemotherapy with the Use of the 21-Gene Test to Manage Early Breast Cancer in a Public Hospital in Brazil | 2021 | Jco Glob Oncol | X | X |
| 91 | McKiernan, J. | Development of a Clinical Implementation Plan (Carepath) for a Novel Urine Exosome Gene Expression Assay as Part of a Two-Cohort, Adaptive Decision Impact Utility Trial | 2018 | J Urology | X |  |
| 92 | McKiernan, J. | Prospective Adaptive Utility Trial to Validate Performance of a Novel Urine Exosome Gene Expression Assay to Predict High-Grade Prostate Cancer in Patients with Prostate-Specific Antigen 2-10 Ng/Ml at Initial Biopsy | 2018 | Eur Urol | X | X |
| 93 | McSorley, L. | Real-World Analysis of Clinical and Economic Impact of 21-Gene Recurrence Score (RS) Testing in Early-Stage Breast Cancer (ESBC) in Ireland | 2020 | J Clin Oncol | X |  |
| 94 | McSorley, L. | Real-World Analysis of Clinical and Economic Impact of 21-Gene Recurrence Score (RS) Testing in Early-Stage Breast Cancer (ESBC) in Ireland | 2021 | Breast Cancer Res Tr | X | X |
| 95 | Meldi, K. | A Prospective, Multi-Center Study to Evaluate the Performance and Clinical Utility of a 15-Gene Expression Profile for Uveal Melanoma. | 2016 | J Clin Oncol | X | X |
| 96 | Michalopoulos, S. | Influence of a Genomic Classifier on Post-Operative Treatment Decisions in High-Risk Prostate Cancer Patients: Results from the Pro-Act Study | 2014 | Curr Med Res Opin | X | X |
| 97 | Michaud, P. | French Prospective Multi-Center Cohort on the Decision Impact Assessment | 2016 | Value Health | X | X |
| 98 | Mouysset, J. | The 21-Gene Assay in the Decision Impact Assessment of ER+, Her2-Breast Cancer: A French Real Life Prospective Study | 2016 | Value Health | X |  |
| 99 | Orucevic, A. | Utilization and Impact of 21-Gene Recurrence Score Assay for Breast Cancer in Clinical Practice Across the United States: Lessons Learned from the 2010 to 2012 National Cancer Data Base Analysis | 2016 | Breast Cancer Res Tr | X | X |
| 100 | O'Shaughnessy, J. | The Breast Cancer Index Registry Study: A Prospective Multi-Center Observational Study to Evaluate Patient Outcome, Clinical Impact, and Medication Adherence in HR Plus Breast Cancer Patients Considering Treatment with Extended Endocrine Therapy | 2022 | Cancer Res. | X | X |
| 101 | Ozmen, V. | Results of the Turkish Prospective Multi-Center Study Utilizing the 21-Gene Oncotype Dx Assay: Decision Impact Analysis. | 2015 | J Clin Oncol | X |  |
| 102 | Ozmen, V. | Impact of Oncotype Dx Recurrence Score on Treatment Decisions: Results of a Prospective Multicenter Study in Turkey | 2016 | Cureus | X | X |
| 103 | Ozmen, V. | Cost Effectiveness of Gene Expression Profiling in Patients with Early-Stage Breast Cancer in A Middle-Income Country, Turkey: Results of A Prospective Multicenter Study | 2019 | Eur J Breast Health | X |  |
| 104 | Pappas, D. | Perceived Clinical Utility of a Test for Predicting Inadequate Response to TNF Inhibitor Therapies in Rheumatoid Arthritis: Results from a Decision Impact Study | 2021 | Rheumatol Int | X | X |
| 105 | Patel, R. | The Informed Genetics Annotated Patient Registry: The Igap Registry | 2021 | Cancer Res | X | X |
| 106 | Petrakova, K. | Decision Impact of the 21-Gene Oncotype Dx Recurrence Score Assay (R) in the Czech Republic on Recommendations for Adjuvant Chemotherapy in Estrogen Receptor Positive Early Stage Breast Cancer (ESBC) Patients | 2019 | Breast | X | X |
| **No.** | **First Author** | **Title** | **Year** | **Journal** | **Included in Bibliometric Analysis** | **Included in Funding Analysis** |
| 107 | Plasseraud, K. | Clinical Performance and Management Outcomes with the Decision Dx-Um Gene Expression Profile Test in a Prospective Multicenter Study | 2016 | J Oncol | X | X |
| 108 | Plun-Favreau, J. | Cost-Effectiveness Analysis of the Use of Oncotype Dx to Guide Adjuvant Chemotherapy Decisions in Breast Cancer Patients in Mexico | 2013 | Value Health | X | X |
| 109 | Rahimi, F. | Rapid Health Technology Assessment of Oncotype Dx in Patients with Early-Stage Breast Cancer | 2022 | Iranian J. Breast Dis. Sociol. | X | X |
| 110 | Raphael, A. | The Impact of Comprehensive Genomic Profiling (CGP) on the Decision-Making Process In The Treatment Of Alk-Rearranged Advanced Non-Small Cell Lung Cancer (ANSCLC) After Failure of 2nd/3rd-Generation Alk Tyrosine Kinase Inhibitors (TKIS) | 2022 | Front. Oncol. | X | X |
| 111 | Reinbolt, R. | Decision Impact Analysis of Comprehensive Genomic Profiling (CGP) in Advanced Breast Cancer: A Prospective Study | 2016 | J Clin Oncol | X | X |
| 112 | Rezai, M. | Impact of the Recurrence Score on Adjuvant Decision-Making in ER-Positive Early Breast Cancer - Results of a Large Prospective Multicentre Decision Impact Study in Node Negative and Node Positive Disease. | 2011 | Cancer Res | X | X |
| 113 | Rodriguez, C. | Impact of the Prosigna (Pam50) Assay on Adjuvant Clinical Decision Making in Patients with Early Stage Breast Cancer: Results of a Prospective Multicenter Public Program | 2017 | J Clin Oncol | X | X |
| 114 | Rosen, B. | Pre-Operative Genomic Profiling for Risk Stratification of Breast Cancer Patients During the Covid-19 Pandemic | 2021 | Ann Surg Oncol | X | X |
| 115 | Rouzier, R. | Evaluation of Intra-Tumor Heterogeneity, Test Reproducibility and their Impact in Breast Cancer Samples Assessed by Prosigna: Results from a Decision Impact Prospective Study and a Matched Case-Control Study. | 2017 | Cancer Res | X | X |
| 116 | Rouzier, R. | Cost-Effectiveness Evaluation of the 21-Gene Breast Cancer Test in France | 2012 | Ann Oncol | X |  |
| 117 | Rouzier, R. | Prospective Multicenter Study of the Impact of the Prosigna Assay on Adjuvant Clinical Decision-Making in Women with Early Stage Breast Cancer: Which Patients are the Best Candidates? | 2016 | J Clin Oncol | X | X |
| 118 | Rouzier, R. | Budget Impact Analysis of the Oncotype Dx (R) Breast Cancer Test in France | 2013 | Breast | X | X |
| 119 | Rouzier, R. | Multigene Assays and Molecular Markers in Breast Cancer: Systematic Review of Health Economic Analyses | 2013 | Breast Cancer Res Tr | X | X |
| 120 | Russell, K. | Multiplatform Tumor Profiling Delivers Value Based Health Care in Refractory Cancer Patients | 2017 | Value Health | X | X |
| 121 | Russell, K. | Treatment Choices Based on Multiplatform Profiling Platform, Unlike Those with Sequencing Alone, Do Not Cause a Cost Explosion in Refractory Cancer Patients | 2017 | Value Health | X | X |
| 122 | Sanft, T. | Prospective Assessment of the Decision-Making Impact of the Breast Cancer Index in Recommending Extended Adjuvant Endocrine Therapy for Patients with Early-Stage ER-Positive Breast Cancer | 2015 | Breast Cancer Res Tr | X | X |
| 123 | Sanft, T. | Prospective Study of the Decision-Making Impact of the Breast Cancer Index in the Selection of Patients with ER Plus Breast Cancer for Extended Endocrine Therapy. | 2015 | J Clin Oncol | X |  |
| 124 | Sanft, T. | Health Economic Impact of Breast Cancer Index (BCI) in Patients with Hormone Responsive Breast Cancer (HRBC) Considering Extended Adjuvant Endocrine Therapy (EET). | 2017 | J Clin Oncol | X |  |
| 125 | Sanft, T. | A Prospective Decision-Impact Study Incorporating Breast Cancer Index into Extended Endocrine Therapy Decision-Making | 2019 | Breast Cancer Manag | X | X |
| 126 | Sanft, T. | Clinical Utility of Biomarker Tests in Decisions on Extended Endocrine Therapy | 2016 | J Clin Oncol | X |  |
| 127 | Sankaran, S. | Canassist Breast Impacting Clinical Treatment Decisions in Early-Stage HR Plus Breast Cancer Patients: Indian Scenario | 2021 | India J Surg Oncol | X | X |
| **No.** | **First Author** | **Title** | **Year** | **Journal** | **Included in Bibliometric Analysis** | **Included in Funding Analysis** |
| 128 | Selmani, Z. | Low Correlation Between Ki67 Assessed by qRT-PCR in Oncotype Dx Score and Ki67 Assessed by Immunohistochemistry | 2022 | Sci Rep | X | X |
| 129 | Sestak, I. | Risk Stratification in Early Breast Cancer in Premenopausal and Postmenopausal Women: Integrating Genomic Assays with Clinicopathological Features | 2019 | Curr Opin Oncol | X | X |
| 130 | Sethi, S. | Percepta Genomic Sequencing Classifier and Decision-Making in Patients with High-Risk Lung Nodules: A Decision Impact Study | 2022 | Bmc Pulm Med | X | X |
| 131 | Sethi, S. | The Impact of a Genomic Sequencing Classifier (GSC) on Clinical Decision Making in Patients with a High-Risk Lung Nodule. | 2021 | J Clin Oncol | X |  |
| 132 | Shivers, S. | Interim Analysis of the Predict Registry: Changes in Treatment Recommendation for a Biologic Signature Predictive of Radiation Therapy (RT) Benefit in Patients with DCIS | 2022 | Cancer Res. | X | X |
| 133 | Smyth, L. | Economic Impact of 21-Gene Recurrence Score Testing on Early Stage Breast Cancer in Ireland | 2015 | Breast | X |  |
| 134 | Smyth, L. | Economic Impact of 21-Gene Recurrence Score Testing on Early-Stage Breast Cancer in Ireland | 2015 | Breast Cancer Res Tr | X | X |
| 135 | Strand, V. | Clinical Utility of Therapy Selection Informed by Predicted Nonresponse to Tumor Necrosis Factor-Alpha Inhibitors: An Analysis from the Study to Accelerate Information of Molecular Signatures (AIMS) in Rheumatoid Arthritis | 2022 | Expert Rev Mol Diagn | X | X |
| 136 | Tharmabala, M. | An Analysis of the Clinical and Economic Impact of the 21-Gene Recurrence Score (RS) in Invasive Lobular Early-Stage Breast Cancer (ESBC) in Ireland | 2021 | Cancer Res | X | X |
| 137 | Thomas, S. | Molecular Profiling with the 92-Gene Assay and Decision-Impact on Cancer Treatment: Interim Results from a Prospective, Multidisciplinary Study. | 2015 | J Clin Oncol | X |  |
| 138 | Thomas, S. | Molecular Diagnosis with the 92-Gene Assay (92-GA) and Decision-Impact on Treatment: Final Results from a Prospective, Multi-Disciplinary Study. | 2016 | J Clin Oncol | X |  |
| 139 | Thomas, S. | Molecular Diagnosis with the 92-Gene Assay (92-GA) and Decision-Impact on Treatment: Final Results from a Prospective, Multi-Disciplinary Study. | 2015 | J Clin Oncol | X |  |
| 140 | Thomas, S. | Multi-Institutional, Prospective Clinical Utility Study Evaluating the Impact of the 92-Gene Assay (Cancertype ID) on Final Diagnosis and Treatment Planning in Patients with Metastatic Cancer with an Unknown or Unclear Diagnosis | 2018 | Jco Precis Oncol | X | X |
| 141 | Torres, S. | Prospective Evaluation of the Impact of the 21-Gene Recurrence Score Assay on Adjuvant Treatment Decisions for Women with Node-Positive Breast Cancer in Ontario, Canada | 2018 | Oncologist | X | X |
| 142 | Tramonti, G. | Decision Impact of a 21-Gene Signature in Early Breast Cancer: A Natural Experiment Using Routine Data | 2018 | Value Health | X | X |
| 143 | Tribedi, T. | P096. An Audit of the Role of Pondx in Chemotherapy Decision-Making in the Breast MDT | 2019 | Ejso-Eur J Surg Onc | X | X |
| 144 | Van Wert, R. | Impact Of a Bronchial Genomic Classifier for Lung Cancer on Reducing Invasive Procedure Recommendations Across Variations in Pulmonology Practices | 2016 | Chest | X | X |
| 145 | Varga, Z. | Summary of Head-to-Head Comparisons of Patient Risk Classifications by the 21-Gene Recurrence Score (R) (RS) Assay and other Genomic Assays for Early Breast Cancer | 2019 | Int J Cancer | X | X |
| 146 | Villarreal-Garza, C. | Change in Therapeutic Management After Endopredict Assay in a Prospective Decision Impact Study of Mexican Premenopausal Patients | 2019 | Cancer Res | X |  |
| 147 | Villarreal-Garza, C. | Change in Therapeutic Management After the Endopredict Assay in a Prospective Decision Impact Study of Mexican Premenopausal Breast Cancer Patients | 2020 | Plos One | X | X |
| 148 | Watanabe, J. | Sunrise-Di Study: Decision Impact of the 12-Gene Recurrence Score (12-R5) Assay on Adjuvant Chemotherapy Recommendation for Stage II and III A/B Colon Cancer | 2019 | Ann Oncol | X | X |
| 149 | Whalen, M. | Prospective Correlation Between Probability of Favorable Pathology on the 17-Gene Genomic Prostate Score and Actual Pathologic Outcomes at Radical Prostatectomy | 2015 | J Urology | X | X |
| **No.** | **First Author** | **Title** | **Year** | **Journal** | **Included in Bibliometric Analysis** | **Included in Funding Analysis** |
| 150 | Whitworth, P. | The Igap Multi-Center Longitudinal Registry Correlating Genetics, Genomics, Imaging, Clinical and Patient-Reported Outcomes | 2021 | Ann Surg Oncol | X | X |
| 151 | Wuerstlein, R. | Results of Multigene Assay (Mammaprint (R)) and Molecular Subtyping (Blueprint (R)) Substantially Impact Treatment Decision Making in Early Breast Cancer: Final Analysis of the WSG Prime Decision Impact Study | 2017 | Cancer Res | X |  |
| 152 | Wuerstlein, R. | Strong Impact of Mammaprint and Blueprint on Treatment Decisions in Luminal Early Breast Cancer: Results of the WSG-Prime Study | 2019 | Breast Cancer Res Tr | X | X |
| 153 | Wuerstlein, R. | Significance of Prospective Multicenter Decision Impact WSG-Bcist Study in Postmenopausal Er+Her2-N0 Early Breast Cancer (EBC) for Molecular Testing for Intrinsic Subtype Definition. | 2015 | J Clin Oncol | X |  |
| 154 | Wuerstlein, R. | The West German Study Group Breast Cancer Intrinsic Subtype Study: A Prospective Multicenter Decision Impact Study Utilizing the Prosigna Assay for Adjuvant Treatment Decision-Making in Estrogen-Receptor-Positive, Her2-Negative Early-Stage Breast Cancer | 2016 | Curr Med Res Opin | X | X |
| 155 | Yamanaka, T. | Prospective Multicenter Study of the Impact of the 12-Gene Assay Recurrence Score on Adjuvant Chemotherapy Treatment Recommendations for Stage II/III Colon Cancer in the Post-Idea Era: Sunrise-Decision Impact Study. | 2018 | J Clin Oncol | X | X |
| 156 | Yamauchi, H. | Prospective Study of the Effect of the 21-Gene Assay on Adjuvant Clinical Decision-Making in Japanese Women with Estrogen Receptor-Positive, Node-Negative, and Node-Positive Breast Cancer | 2014 | Clin Breast Cancer | X | X |
| 157 | Yamauchi, H. | Decision Impact and Economic Evaluation of the 21-Gene Recurrence Score (Rs) Assay for Physicians and Patients in Japan | 2011 | Eur J Cancer | X |  |
| 158 | Yamauchi, H. | Societal Economics of the 21-Gene Recurrence Score (R) in Estrogen Receptor-Positive Early-Stage Breast Cancer in Japan | 2012 | Cancer Res | X |  |
| 159 | Yordanova, M. | The Role of the 21-Gene Recurrence Score(R) Assay in Hormone Receptor-Positive, Node-Positive Breast Cancer: The Canadian Experience | 2022 | Curr. Oncol. | X | X |
| 160 | Zambelli, A. | Prospective Observational Study on the Impact of the 21-Gene Assay on Treatment Decisions and Resources Optimization in Breast Cancer Patients in Lombardy: The Bondx Study | 2020 | Breast | X | X |
| 161 | Zanotti, L. | Diagnostic Tests Based on Gene Expression Profile in Breast Cancer: From Background to Clinical Use | 2014 | Tumor Biol | X | X |
| 162 | Zhang, S. | Oncotype Dx Recurrence Score in Premenopausal Women | 2022 | Ther. Adv. Med. Oncol. | X | X |
| 163 | Zissiadis, Y. | The Predict Registry Australia: A Prospective Registry Study to Evaluate the Clinical Utility of The Dcisionrt Test on Treatment Decisions in Patients with DCIS Following Breast Conserving Surgery | 2022 | Cancer Res. | X | X |
