# Supplementary material for: Industry involvement in evidence production for genomic medicine: A bibliometric and funding analysis of decision impact studies
Source: PLoS One. 2023 Apr 27;18(4):e0285122. doi: 10.1371/journal.pone.0285122 (PMC10138259; doi:10.1371/journal.pone.0285122)
Supplement: Attachment — Submitted filename: Response to Reviewers.pdf [file pone.0285122.s007.pdf]

**Industry involvement in evidence production for genomic medicine: A bibliometric and funding analysis of decision impact studies**

Dear Dr. Chenette,

Thank you for the invitation to revise and resubmit our manuscript. For the actionable items, we have addressed the reviewer's comments. We do need to state that we were disappointed with the quality of the review. It appears that Reviewer One has sent comments on another manuscript, as their comments to not apply to our manuscript and Reviewer Two incorrectly referenced figures and provided a vague comment that cannot be actioned. Responses to the comments may be found in the table below.

Sincerely,

Gillian Parker

| | Reviewer Comment | Action |
|---|---|---|
| **Reviewer One** | | |
| *1* | *I would suggest authors to create/develop Research questions that this review is aimed to answer. Authro can refer the following papers for development of the RQs* | Our manuscript includes clearly stated research questions. The research questions, rationale and study objectives are detailed lines 153-165. |
| *2* | *Once you developed RQs you can use these RQs for the organisng your results and presneting them so that readers can be benefited. Also please add indigshts to each analysis what it means before your report what you found in anlysis. Conclusion can be strengthened by highlighting major implications of findings* | Our research questions were detailed in the background section (lines 153-165) and then used to organize and present our results. The results presented respond to the inquiries in the research questions.<br>The Discussion section provides detailed insights, analysis and implications of findings. |
| *3* | *What next ? Based on your findings can you propose some future reseach questions that future scholars can use to pursue research in the filed.* | Our directions for future research section is clearly delineated by the bolded heading DIRECTIONS FOR FUTURE RESEARCH and text (lines 628-636). |
| **Reviewer Two** | | |
| *4* | *Tell readers and provide code in R for drawing Figure 2 and 8-10.* | Figures 2, 8-10 were not generated using R code. (All of our figures have detailed legends noting the sources used for analysis and visualization, as stated in the legends.) Figure 3 was generated using R code, so we assume that is the figure the Reviewer is referencing. We have included a supplementary file with the R code for this figure (S4 Appendix) and noted this inclusion in the manuscript (lines 290 – 291). |
| 5 | *Study data should provided with a textfile that meeting the study under the biblioshiny app in biblimetrics* | We have included 2 supplementary files:<br>1. Metadata for Biblioshiny (S2 Appendix)<br>2. Metadata for Funding Analysis (analysis done in Excel; S3 Appendix)<br>And noted these inclusions in the manuscript (lines 270 – 271). |
| 6 | *Two sections in introduciton and discussions should be rewritten for readers to pursuit the authors' tempo in this study.* | This comment is not actionable. |

**Industry involvement in evidence production for genomic medicine: A bibliometric and funding analysis of decision impact studies**

| | **Additional changes** | |
|---|---|---|
| *7* | Change Corresponding Author | Please note the corresponding author has been changed in the manuscript – from Gillian Parker to Fiona A. Miller.<br>Additionally, we are requesting that this change be made in the online system for correspondence. |
| *8* | Add all Author's ORCID IDs | Please note that we would like to include ORCID IDs for all authors:<br><br>Gillian Parker: 0000-0001-9884-9181<br><br>Sarah Hunter: 0000-0002-5346-8382<br><br>Stuart Hogarth: 0000-0001-6796-8815<br><br>Fiona Miller: 0000-0003-4953-6255 |
| **Please note: the additional items listed below relate to very minor word/phrasing changes; they are not substantive** | | |
| *9* | Change to description of funding analysis object of interest | Please note that we have corrected the terminology used to describe the object of interest in the funding analysis on line 216—from "unit of analysis" to "unit of observation".<br><br>Since submitting this manuscript, we have done further work on bibliometric methods; we used this term in error, and we have corrected it. |
| *10* | Changes to ensure the consistent application of the plural form of analyses, when referring to bibliometric and funding analyses | Please note we have ensured that we have consistently referred to the analysis-types throughout in the plural ("bibliometric and funding analyses") instead of the singular ("bibliometric and funding analysis").<br><br>Upon re-reading our manuscript we noted these inconsistencies and corrected them. |
| *11* | Changes to the units of analysis identified in Figures 4-10 | Please note that the following corrections have been made to the figures listed below:<br>• **Fig 4**—description of unit of analysis change from 'publication', to: 'author'<br>• **Fig 5**—description of unit of analysis change from 'publication', to: 'author affiliation'<br>• **Fig 6**—description of unit of analysis change from 'publication', to: 'pairs of author collaboration'<br>• **Fig 7**—description of unit of analysis change from 'author affiliation country', to: 'pairs of country collaboration'<br>• **Fig 8**—description of unit of analysis change from 'publication', to: 'funding status'<br>• **Fig 9**—description of unit of analysis change from 'publication', to: 'disclosure status'<br>• **Fig 10**—description of unit of analysis description change from 'publication', to: 'author affiliation' |

**Industry involvement in evidence production for genomic medicine: A bibliometric and funding analysis of decision impact studies**

| | | |
|---|---|---|
| | | As previously described, since submitting this manuscript, we have done further work on bibliometric methods; we used the term "unit of analysis" in error, and we have corrected this.<br><br>Please note we have uploaded new figure files with the correct terminology (in other words, the changes to these figures have not been submitted in tracked changes) |
| *12* | Change to the software listed in Figure 1 | Please note 'wordcloud' was listed as a software tool used to produce the visualizations under the 'Data Visualization' section of the diagram.<br><br>Upon re-reading our manuscript, we noted that we had omitted listing this software tool in this diagram, and we corrected this.<br><br>Please note: we have uploaded a corrected version of this figure. |
