# Supplementary material for: Industry involvement in evidence production for genomic medicine: A bibliometric and funding analysis of decision impact studies
Source: PLoS One. 2023 Apr 27;18(4):e0285122. doi: 10.1371/journal.pone.0285122 (PMC10138259; doi:10.1371/journal.pone.0285122)
Supplement: Attachment — Submitted filename: Abstract.docx [file pone.0285122.s006.docx]

The manuscript shows promise, but there are certain limitations that require clarification and revision in accordance with my feedback outlined below. I trust that these enhancements will enhance the readability and comprehensiveness of the manuscript for the benefit of its readers.

1. Abstract:

Overall, the abstract provides a clear and concise summary of the study's background, methods, and results. The authors effectively communicate the purpose of the study, which is to analyze the origins and intentions of decision impact studies in genomic medicine, with a focus on the involvement of industry actors.

The use of bibliometric and funding analyses is appropriate for this type of study and the results are presented in a straightforward manner. The finding that decision impact studies are primarily produced for proprietary genomic assays for use in cancer care and are funded by industry is particularly noteworthy.

One suggestion for improvement would be to provide more information about the specific databases searched and inclusion criteria used for the study(e.g., How to draw Figure 2 for readers who are able to draw it on their own in the future). Additionally, it would be helpful to include a sentence or two on the implications of these findings for the field of genomic medicine and for patients, particularly regarding the need for further research into the use of these studies in decision-making for coverage and reimbursement.

Overall, the abstract effectively communicates the study's purpose, methods, and findings and provides important insights into the role of industry in the production of evidence in genomic medicine.

2. Introduction

Prior to line 150, the motivation linked to the study objectives should be rewritten in make readers easy to capture the reasons of importance and the use of bibliometrics that deserve reading the manuscript. That is, more constructed and solid transitions are required for readers to know.

3. METHODS

Lines 169 to 178, those introductions to bibliometrics are required in Introduction section.

In line 244, an easy-to-use interface (Biblioshiny) was performed. A good research should provide original dataset for readers who are able to replicate the study. We have not seen the data provided in this study. If readers who possess the data, the Biblioshiny can be applied to generate the results as did in this study.

In lines 265 to 296, we can perform the biblioshiny to gain the results described in this section. Some definitions should be clarified, such as what are the node size in network analysis. Any difference in method(e.g., the bubble size) exists between author collaborations and keyword analysis or citation analysis for cited references.

How to draw Figure 2. Whether the diagram was drawn in biblioshiny or not. If not, what is the code in R used to draw the Figure. The methods should be expressed in Methods

Results:

Word cloud was used in Figure 3. What is the reason for using word cloud rather than using co-word analysis by clustering the keywords(or keywords plus) that should be mentioned in Methods.

Figures 4 to 7 were drawn in biblioshony. Why the bubble sizes in Figure 7 are equal Otherwise, we cannot know which countries are the leaders in clusters. Next, what is the criterion that was used to determine the bubble size if bubble size is computed. If so, Table 1 in line 410 can be omitted(Top 20 Affiliates, Country of Origin), but only five are shown in Table 1 instead of 20.

The labels in Figure 6 are too small. It is necessary to enlarge the label size for easily to know each one is represented by which keyword. E.g., then reproduced (using Adobe Illustrator) to provide clarification or 294 additional analysis in line 293.

It is better to know the R-code regarding Figures 8 to 10 deposited in manuscript for readers who can replicate the drawing in the future study.

Discussion

1. In Introduction , we see three points should be verified in this study. In DIRECTIONS FOR FUTURE RESEARCH, we see four items that are highlighted. I s there any difference of inconsistence between the two. W are going to expect the consistence between the two prior and post scenarios.
2. In STRENGTHS AND LIMITATIONS, we thought that many studied should be conducted with the same or similar altitude and spirit in those ways mentioned by the authors. Similarly, the limitations are also not touched much on the drawbacks or limitations that should be noticed for future studies.

If possible, the biblioshiny can be mentioned and compared with other software used for bibliometrics. Furthermore, the R used in bibliometrics can be mentioned also in biblimetrics when compared to others using traditional software to report similar reports.

3. Other parts in Discussions, we are not interested in the findings due to much spaced stuffed in this parts. I suggested the authors highlight some subtitle such as main findings, similar results in literature, what have been added to future research from this study and implications.
